# Genetic causal relationship between age at menarche and benign oesophageal neoplasia identified by a Mendelian randomization study

**DOI:** 10.3389/fendo.2023.1113765

**Published:** 2023-03-21

**Authors:** Yani Su, Yunfeng Hu, Yiwei Xu, Mingyi Yang, Fangcai Wu, Yuhui Peng

**Affiliations:** ^1^ Department of Clinical Laboratory Medicine, Cancer Hospital of Shantou University Medical College, Shantou, China; ^2^ Esophageal Cancer Prevention and Control Research Center, Cancer Hospital of Shantou University Medical College, Shantou, China; ^3^ Guangdong Esophageal Cancer Institute, Guangzhou, China; ^4^ Department of Radiotherapy, Yan'an University Affiliated Hospital, Yan’an, China; ^5^ Department of Joint Surgery, Honghui Hospital, Xi’an Jiaotong University, Xi’an, China; ^6^ Department of Radiation Oncology, Cancer Hospital of Shantou University Medical College, Shantou, China

**Keywords:** hormone, age at menopause, instrumental variables, confounding factor, genetic

## Abstract

**Objective:**

The occurrence and development of oesophageal neoplasia (ON) is closely related to hormone changes. The aim of this study was to investigate the causal relationships between age at menarche (AAMA) or age at menopause (AAMO) and benign oesophageal neoplasia (BON) or malignant oesophageal neoplasia (MON) from a genetic perspective.

**Methods:**

Genome-wide association study (GWAS) summary data of exposures (AAMA and AAMO) and outcomes (BON and MON) were obtained from the IEU OpenGWAS database. We performed a two-sample Mendelian randomization (MR) study between them. The inverse variance weighted (IVW) was used as the main analysis method, while the MR Egger, weighted median, simple mode, and weighted mode were supplementary methods. The maximum likelihood, penalized weighted median, and IVW (fixed effects) were validation methods. We used Cochran’s Q statistic and Rucker’s Q statistic to detect heterogeneity. The intercept test of the MR Egger and global test of MR pleiotropy residual sum and outlier (MR-PRESSO) were used to detect horizontal pleiotropy, and the distortion test of the MR-PRESSO analysis was used to detect outliers. The leave-one-out analysis was used to detect whether the MR analysis was affected by single nucleotide polymorphisms (SNPs). In addition, the MR robust adjusted profile score (MR-RAPS) method was used to assess the robustness of MR analysis.

**Results:**

The random-effects IVW results showed that AAMA had a negative genetic causal relationship with BON (odds ratio [OR] = 0.285 [95% confidence interval [CI]: 0.130-0.623], *P* = 0.002). The weighted median, maximum likelihood, penalized weighted median, and IVW (fixed effects) were consistent with random-effects IVW (*P* < 0.05). The MR Egger, simple mode and weighted mode results showed that AAMA had no genetic causal relationship with BON (*P* > 0.05). However, there were no causal genetic relationships between AAMA and MON (OR = 1.132 [95%CI: 0.621-2.063], *P* = 0.685), AAMO and BON (OR = 0.989 [95%CI: 0.755-1.296], *P* = 0.935), or AAMO and MON (OR = 1.129 [95%CI: 0.938-1.359], *P* = 0.200). The MR Egger, weighted median, simple mode, weighted mode, maximum likelihood, penalized weighted median, and IVW (fixed effects) were consistent with a random-effects IVW (*P* > 0.05). MR analysis results showed no heterogeneity, the horizontal pleiotropy and outliers (*P* > 0.05). They were not driven by a single SNP, and were normally distributed (*P* > 0.05).

**Conclusion:**

Only AAMA has a negative genetic causal relationship with BON, and no genetic causal relationships exist between AAMA and MON, AAMO and BON, or AAMO and MON. However, it cannot be ruled out that they are related at other levels besides genetics.

## 1 Introduction

Oesophageal neoplasia (ON) is a major health problem worldwide, and its incidence rate is rising rapidly (1, 2). ON can be divided into benign oesophageal neoplasia (BON) and malignant oesophageal neoplasia (MON). About 80% of ON is malignant (3). MON is a common cause of cancer mortality, especially squamous cell carcinoma and adenocarcinoma (3). BON, while uncommon, can cause sudden death with difficulty swallowing (4). Surgery, chemotherapy, radiotherapy, molecular targeted therapy, and their combinations are currently available treatment options for ON (2, 5). However, the prognosis of MON is still quite dismal, despite therapeutic advances (6), and it continues to pose a severe threat to human health (7, 8).

According to their location on the esophageal wall, BONs can be divided into intramural neoplasms and luminal neoplasms. Luminal neoplasms account for nearly one third of BONs (9). Esophageal leiomyoma is one of the most common BONs, accounting for more than 50%. Compared with cancer, most patients with fibroids have no symptoms, and the most common form imaged is an intramural submucosal mass located in the middle and lower part of the esophagus, with smooth muscle arrangement in part of the esophagus (10). A variety of BONs may require esophagectomy, including esophageal leiomyomas, gastrointestinal stromal tumors, schwannomas, granular cell tumors, inflammatory pseudotumors, hemangioma, adenoma, fibrovascular polyp, and extraesophageal tumors that invade or constrict the esophagus. Among them, leiomyoma is the most common BON requiring esophagectomy, and occurs in about 10% of cases (9).

MON is one of the most common cancers in the world, ranking seventh in incidence (11, 12). Risk factors include smoking, alcohol consumption, low fruit intake and high body mass index, and it is becoming a major disease burden worldwide (12). MONs are mainly divided into esophageal squamous cell carcinoma (ESCC) and esophageal adenocarcinoma (EAC) (6). Barrett’s esophagus is a precancerous lesion that can progress to EAC (13). EAC is most commonly found in developed countries (such as Europe and the United States), whereas ESCC mainly occurs in developing countries, including Africa and East Asia, especially in China (14). In recent decades, due to advances in multidisciplinary diagnosis and treatment, the overall survival rate of MON has been greatly improved, but the effect is still unsatisfactory (11, 15). Therefore, it is imperative to continue to search for risk factors of MON.

Age at menarche (AAMA) and age at menopause (AAMO) have been shown to be risk factors for a variety of diseases. A younger AAMA and later AAMO are strongly associated with increased risk of breast cancer and endometrial cancer, respectively (16, 17). Studies found that later AAMA is linked with reduced risk of coronary artery disease and higher risk of chronic kidney disease (18, 19). Moreover, later AAMO is associated with an increased risk of cardiovascular disease (18, 20), as well as an increased risk of lung cancer (21). More importantly, hormone therapy during menopause has been shown to reduce the risk of EAC in women (22). A prior study found that reproductive factors are associated with ON risk (23). A clinical reproductive prognosis model with AAMO has good prognostic value in predicting the overall survival of female Chinese ESCC patients (24), and AAMO and hormone replacement therapy have been shown to be risk factors for ESCC (25). However, the correlation between AAMA, AAMO and ON is not clear. Thus, there is a strong need to investigate the correlation between AAMA, AAMO and ON.

According to Mendelian laws of heredity, germline genetic information is randomly fixed at conception, and Mendelian randomization (MR) methods use genetic variations, such as single nucleotide polymorphisms (SNPs), as instrumental variables to assess genetic causality between traits and diseases or between diseases (26). MR analysis is not affected by confounding factors and reverse causality of traditional epidemiological methods, such as retrospective studies, and has been widely used (27). MR analysis has previously been used to find a genetic causal relationship between AAMA and osteoporosis (28), and a meta-analysis and MR study has found a causal association between AAMA and epithelial ovarian cancer risk (29). In addition, another MR study found a causal association between AAMA, AAMO and risk of colorectal cancer (30). Here, we examine the genetic causal relationship between AAMA and AAMO, and outcome (BON and MON) at the genetic level through the MR analysis.

## 2 Materials and methods

### 2.1 Study design

This study was based on a genome-wide association study (GWAS) summary data of exposures (AAMA and AAMO) and outcomes (BON and MON), and selected eligible SNPs as instrumental variables for MR analysis to investigate the genetic causal relationship between them. This study strictly followed the three assumptions of MR analysis: 1) the selected instrumental variables were strongly correlated with exposures (*P* < 5×10^-8^ and F statistic > 10); 2) The selected instrumental variables were not associated with any confounders that affected the association between exposures and outcomes; 3) the selected instrumental variables affected the outcome only through exposure, but not through other pathways. All datasets used in this study are publicly available. Ethical permission and written informed consent had been provided in the initial studies. Details of the data used in this study are shown in **Supplementary Table 1**.

### 2.2 GWAS summary data of AAMA and AAMO

GWAS summary data of AAMA and AAMO were obtained from the IEU OpenGWAS database (https://gwas.mrcieu.ac.uk/). GWAS summary data of AAMA comprised 182,416 female samples and 2,441,816 SNPs, while data of AAMO comprised 69,360 female samples and 2,418,696 SNPs. All samples were of European descent and informed consent was provided (31, 32). In short, the studies were asked to use the full imputed set of HapMap Phase 2 autosomal SNPs, and to run an additive model including top principal components and study specific covariates. In some cases, studies submitted data using 1000 genomes–based imputation. Cases without SNPs in the HapMap 2 set were removed. Once data was submitted, each study was quality controlled centrally according to standard quality control protocols independently by two analysts. SNPs were filtered out if the minor allele frequency (MAF) was less than 1%, or if the imputation quality metrics were low (imputation quality < 0.4). Studies and SNPs passing quality control were combined using an inverse-variance weighted meta-analysis, implemented using METAL (33). The PLINK clumping commands were used to identify the most significant SNPs in associated regions, using only those SNPs that had data from more than 50% of the studies (34). SNPs were considered as having genome-wide significance only if *P* < 5×10^−8^. Detailed information of the data can be found in a previous study (31, 32).

### 2.3 GWAS summary data of BON and MON

GWAS summary data for BON and MON was obtained from the IEU OpenGWAS database (https://gwas.mrcieu.ac.uk/). GWAS summary data for BON was comprised of 144 cases and 218,648 controls (males and females) and contained 16,380,466 SNPs, whereas data for MON was comprised of 232 cases and 218,560 controls (males and females) and contained 16,380,466 SNPs. All participants were of European descent and provided informed consent. GWAS summary data for BON and MON were produced by the FinnGen consortium. The FinnGen research project is a public-private partnership that integrates disease endpoint genetic data provided by the Finnish Biobank and the Finnish Health Registry (35). The FinnGen research project aims to identify genotype-phenotype correlations in the Finnish population. All cases were defined using the M13 code in the International Classification of Diseases-Tenth Revision (ICD-10). These individuals were genotyped using Illumina (Illumina Inc, San Diego) and Affymetrix chip arrays (Thermo Fisher Scientific, Santa Clara, CA, USA). Detailed information on the participants, genotyping, imputation, and quality control can be found on the FinnGen website (https://finngen.gitbook.io/documentation/).

### 2.4 Selection of instrumental variables

In order to ensure the robustness of MR analysis results, we screened qualified SNPs as instrumental variables through a series of strict quality controls, and performed MR analysis of exposures and outcomes. First, we obtained SNPs associated with exposures (*P* < 5×10^-8^). Second, since strong linkage disequilibrium among the selected SNPs could lead to biased results, the clumping process (r^2^ < 0.001, clumping distance = 10,000 kb) was carried out to eliminate the linkage disequilibrium between the included instrumental variables (35). Third, those SNPs associated with outcomes (*P* < 5×10^-8^) were not included in the instrumental variables. Fourth, we applied the PhenoScanner database (http://www.phenoscanner.medschl.cam.ac.uk/phenoscanner) to delete SNPs that were associated with confounding factors (36). From previous literature and studies, we found that the main risk factors for outcomes were smoking, alcohol and obesity (37, 38). Fifth, to satisfy the strong association with exposure, we selected SNPs with an F statistic > 10 as instrumental variables. F statistics were calculated using the formula: F=R^2^(N-K-1)/K(1-R^2^). R^2^ was calculated using the formula: R^2 =^ 2×MAF×(1-MAF) Beta^2^ (39). Sixth, palindromic SNPs with intermediate allele frequencies were excluded to guarantee that the impact of SNPs on exposures corresponded to the same allele as that providing the effect on outcomes (40).

### 2.5 Statistical analysis

The *TwoSampleMR* and *MRPRESSO* packages of R (version 4.1.2) were used to perform two-sample MR analyses of exposures (AAMA and AAMO) and outcomes (BON and MON). The random-effects inverse variance weighted (IVW) was used as the main analytical method, while MR Egger, weighted median, simple mode, and weighted mode were used as supplementary methods. Our MR analysis results followed the results of the random-effects IVW (40–42). The random-effects IVW allowed for each SNP to have different mean effects (43). The IVW method uses a meta-analysis approach to combine the Wald ratio estimates of the causal effect obtained from different SNPs, and provides a consistent estimate of the causal effect of the exposures on the outcomes when each genetic variant satisfies the assumptions of an instrumental variable (44). The MR Egger method is able to assess whether genetic variants have pleiotropic effects on the outcomes (45). Weighted median analysis serves as an important method of estimating the causal effect if over 50% of SNPs meet the “no horizontal pleiotropy” assumption (46). The simple mode is a model-based estimation method that provides the robustness for pleiotropy (47). The weighted mode is sensitive to the difficult bandwidth selection for mode estimation (48).

The Cochran’s Q statistic of the MR-IVW method, and Rucker’s Q statistic of the MR Egger method were used to determine the heterogeneity of MR analysis, where *P* > 0.05 indicates no heterogeneity (49). The intercept test of MR Egger and global test of MR pleiotropy residual sum and outlier (MR-PRESSO) were used to detect horizontal pleiotropy, where *P* > 0.05 indicates no horizontal pleiotropy (36). The distortion test of MR-PRESSO analysis was used to detect whether the MR analysis results were affected by outliers (50). If there were outliers, a second round of MR analysis was performed after the outliers were removed. Leave-one-out analysis was used to investigate whether the genetic causal relationship between exposures and outcomes was influenced by a single SNP (44). If the MR analysis results were affected by a single SNP, in order to prevent the occurrence of false positives or false negatives to the greatest extent, we carried out a second round of genetic assessment after deleting the single SNP that affected the MR analysis results. Moreover, the MR robust adjusted profile score (MR-RAPS) method was used to validate the robustness of the MR analysis (26). A *P* > 0.05 indicated that it conformed to the normal distribution and the evaluation results had strong robustness. Finally, the maximum likelihood, penalized weighted median, and IVW (fixed effects) were used as validation methods for the genetic causal relationship between exposures and outcomes.

## 3 Results

### 3.1 Selection of instrumental variables

Through screening for SNPs associated with exposures (*P* < 5×10^-8^) and removing the linkage disequilibrium, AAMA and AAMO yielded 68 and 42 SNPs, respectively. We further identified 68 SNPs shared by AAMA and BON, and found that there were no SNPs associated with BON. After excluding seven SNPs associated with confounding factors, we used the remaining 61 SNPs as instrumental variables (F statistic >10), in which there were seven palindromic SNPs: rs9373571, rs4801589, rs4242496, rs2836950, rs1874984, rs1518080 and rs11756454 (**Supplementary Table 2**). Similarly, we identified 68 SNPs shared by AAMA and MON, and found that there were no SNPs associated with MON, except for seven SNPs associated with confounding factors, 61 SNPs were used as instrumental variables (F statistic >10), and there were seven palindromic SNPs: rs9373571, rs4801589, rs4242496, rs2836950, rs1874984, rs1518080 and rs11756454 (**Supplementary Table 3**).

We also analyzed 41 SNPs shared by AAMO and BON, and found that there were no SNPs associated with BON. Except for two SNPs associated with confounding factors, the remaining 39 SNPs were used as instrumental variables (F statistic >10), and there were three palindromic SNPs: rs1054875, rs12599106 and rs2236918 (**Supplementary Table 4**). We further identified 41 SNPs shared by AAMO and MON, and found that there were no SNPs associated with MON. After excluding two SNPs associated with confounding factors, we used the remaining 39 SNPs as instrumental variables (F statistic >10), and there were three palindromic SNPs: rs1054875, rs12599106 and rs2236918 (**Supplementary Table 5**).

Because the effect of palindromic SNPs on exposure may not correspond to the alleles that influence outcomes, the palindromic SNPs were excluded from the MR analysis. Ultimately, 54 SNPs were used in the AAMA MR analysis and 36 SNPs in the AAMO MR analysis.

### 3.2 MR analysis of AAMA, AAMO and BON

**Figure 1:**
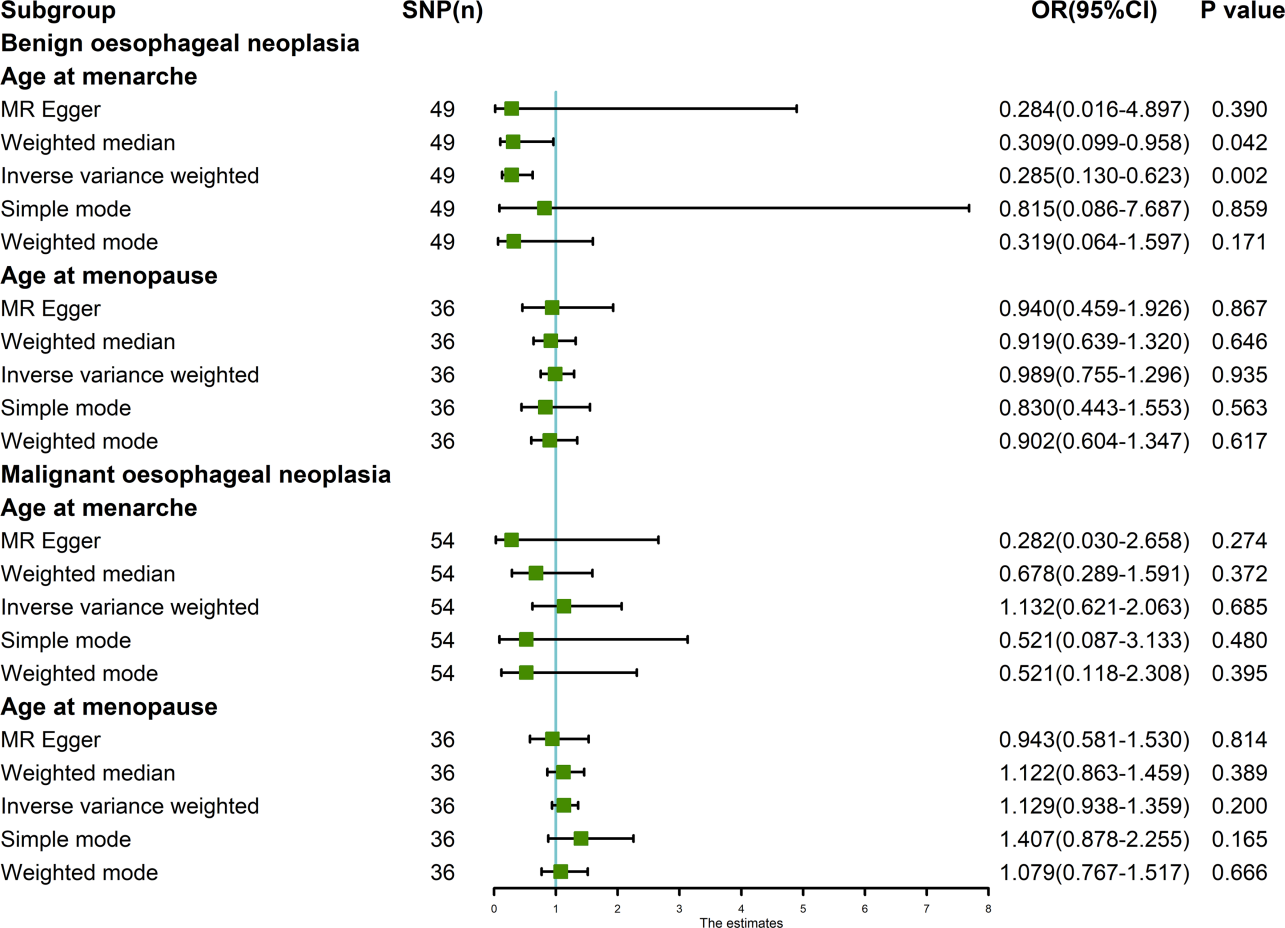
MR analysis of exposures (age at menarche and age at menopause) and outcomes (benign oesophageal neoplasia and malignant oesophageal neoplasia). Five methods: random-effects IVW, MR Egger, weighted median, simple mode, and weighted mode. Our MR analysis follows the results of random-effects IVW. The blue vertical line represents OR = 1. The green square is the OR value of the MR analysis result. The black line segment is the OR 95% confidence interval.

**Figure 2:**
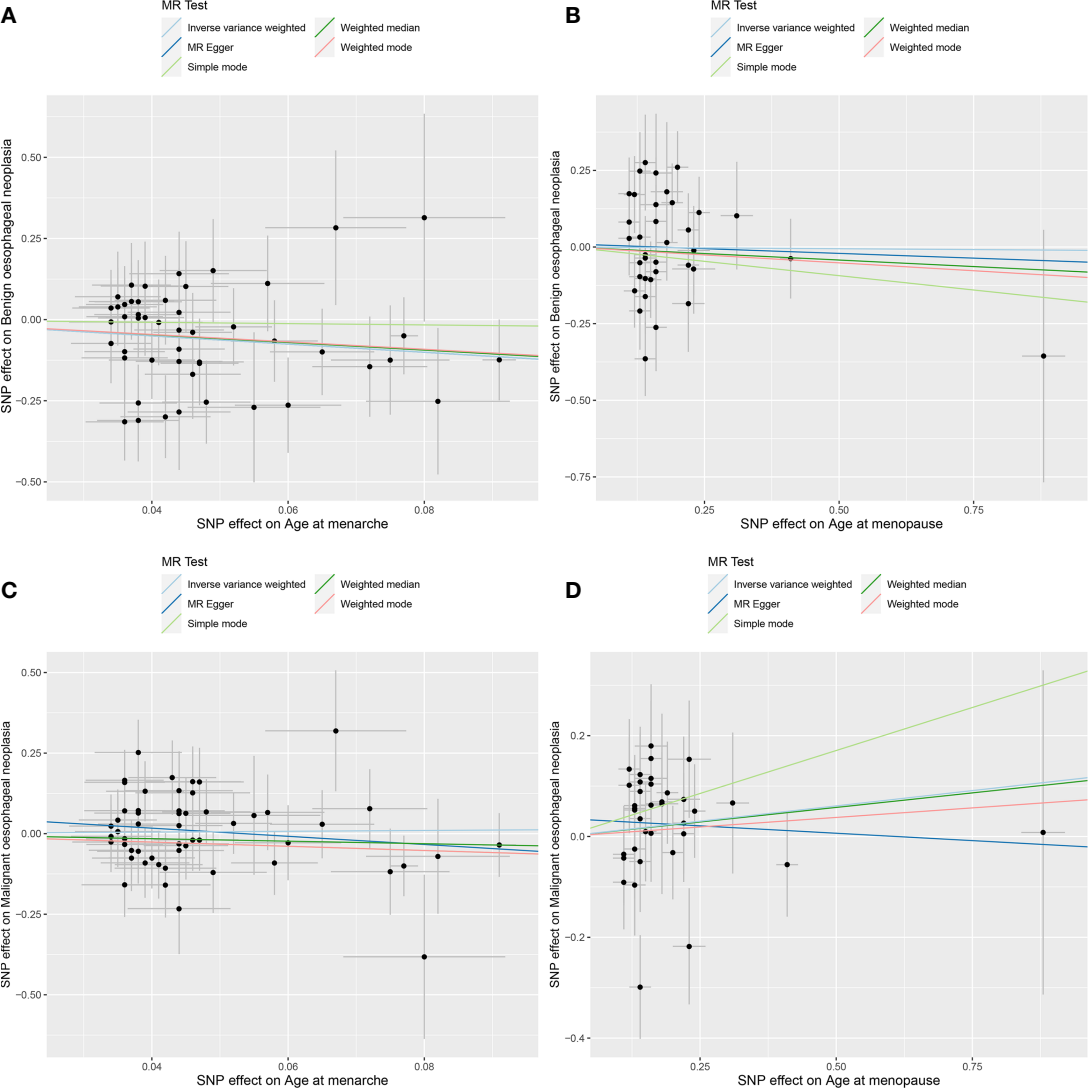
MR analysis scatter plot. Different colored lines in the figure represent the results of the different MR analysis methods. Oblique upward indicates positive causality, oblique downward indicates negative causality. **(A)** age at menarche and benign oesophageal neoplasia; **(B)** age at menopause and benign oesophageal neoplasia; **(C)** age at menarche and malignant oesophageal neoplasia; **(D)** age at menopause and malignant oesophageal neoplasia.

**Figure 3:**
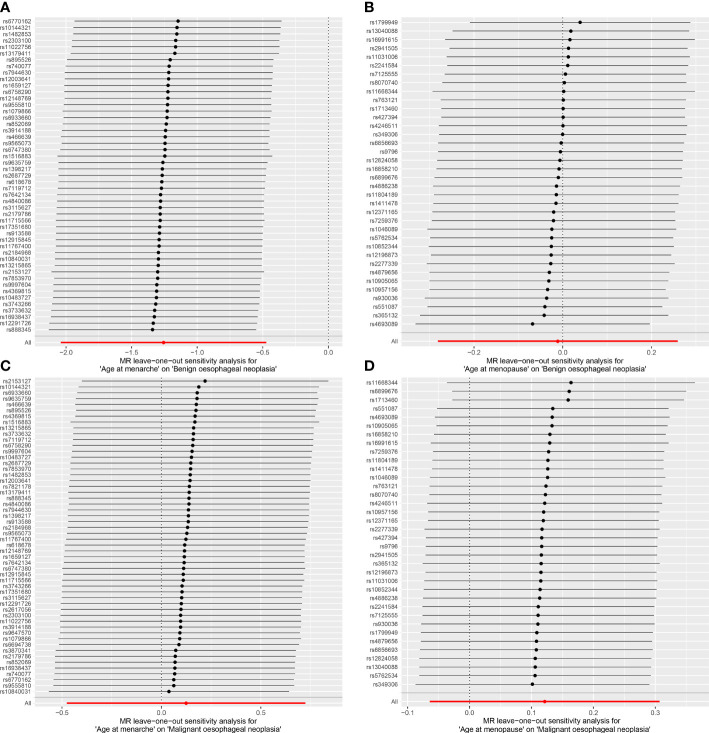
Leave-one-out analysis of the results from MR analysis. Each black line in the figure refers to the result of MR analysis with the remaining SNPs after deleting one SNP on the left. **(A)** age at menarche and benign oesophageal neoplasia; **(B)** age at menopause and benign oesophageal neoplasia; **(C)** age at menarche and malignant oesophageal neoplasia; **(D)** age at menopause and malignant oesophageal neoplasia.

**Figure 4:**
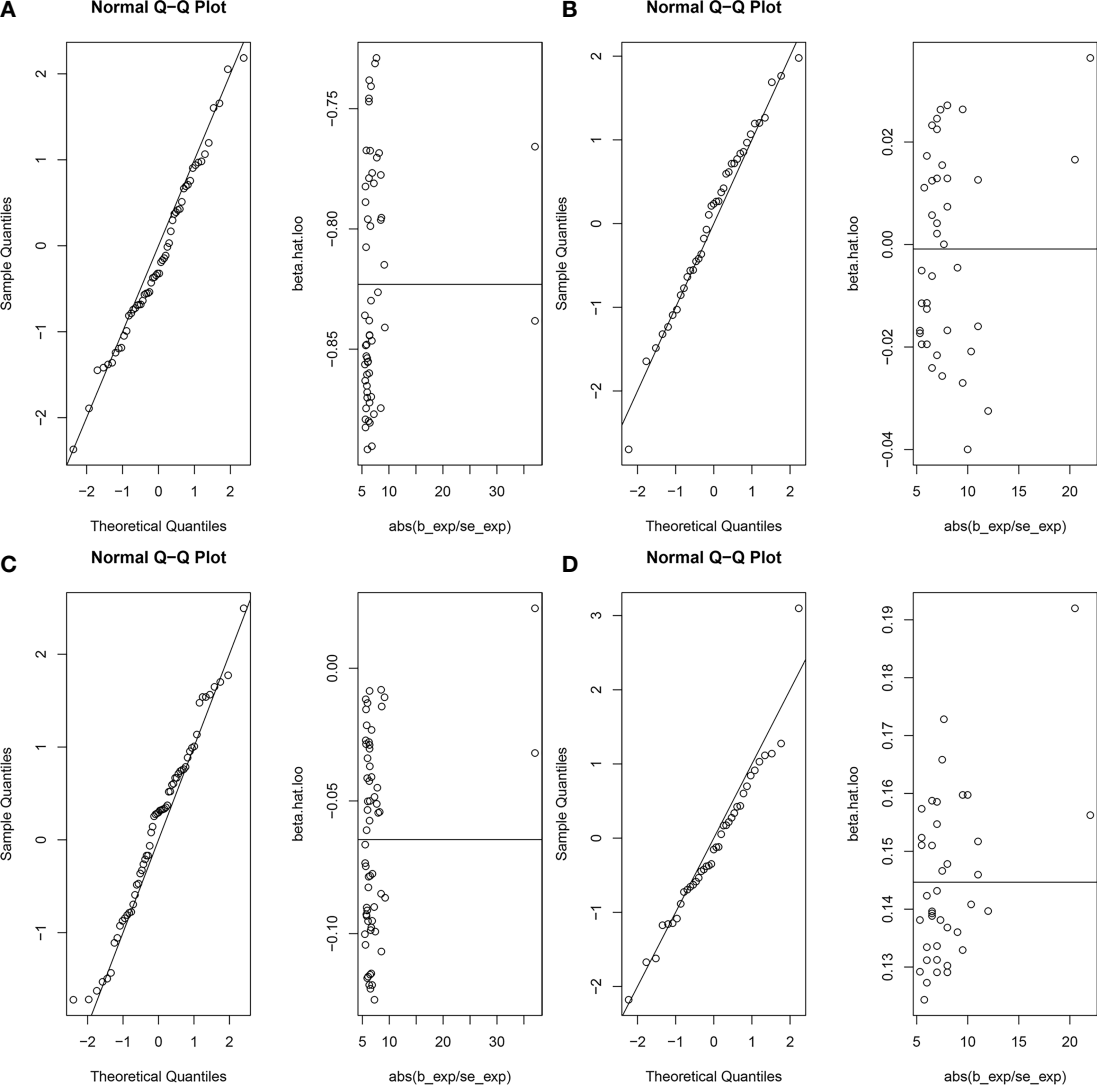
Normal distribution plots of the MR analysis. Circles in the figure represent SNPs for MR Analysis. **(A)** age at menarche and benign oesophageal neoplasia; **(B)** age at menopause and benign oesophageal neoplasia; **(C)** age at menarche and malignant oesophageal neoplasia; **(D)** age at menopause and malignant oesophageal neoplasia.

The random-effects IVW results showed that AAMA had no genetic causal relationship with BON (odds ratio [OR] = 0.457 [95% confidence interval [CI]: 0.195-1.071], *P* = 0.072) (**Supplementary Figures 1A, B**). The Cochran’s Q statistic of the MR-IVW method (*P* = 0.090), and Rucker’s Q statistic of the MR Egger method (*P* = 0.081) showed that the MR analysis of AAMA and BON had no heterogeneity. The intercept test of MR Egger (*P* = 0.538) and global test of MR-PRESSO (*P* = 0.090) showed that the MR analysis of AAMA and BON had no horizontal pleiotropy. The distortion test of MR-PRESSO analysis showed that the MR analysis of AAMA and BON had no outliers. However, “leave-one-out” analysis indicated that the MR analysis of AAMA and BON was driven by a single SNP (five SNPs) (**Supplementary Figure 1B**). We performed a preliminary MR analysis of AAMA and BON using 54 SNPs. As a result of the leave-one-out analysis, a second round of MR analysis was performed after five SNPs were removed. A follow-up analysis was conducted for the remaining 49 SNPs. The second round of random-effects IVW results showed that AAMA had a negative genetic causal relationship with BON (OR = 0.285 [95%CI: 0.130-0.623], *P* = 0.002) (**Figures 1**, **2A**). The analysis results of weighted median were consistent with random-effects IVW, and MR Egger, simple mode, and weighted mode showed that AAMA had no genetic causal relationship with BON (**Figure 1**). There was no heterogeneity and horizontal pleiotropy (*P* > 0.05), and there were no outliers (**Table 1**). The leave-one-out analysis indicated that the MR analysis of AAMA and BON was not driven by a single SNP (**Figure 3A**), and the MR-RAPS analysis showed that the MR analysis between AAMA and BON was normally distributed (*P* > 0.05) (**Table 1**; **Figure 4A**).

**Table 1:** Sensitivity analysis of the MR analysis results of exposures and outcomes.

Exposure	Outcome	Heterogeneity Test		Pleiotropy test	MR-PRESSO		MR-RAPS
		Cochran’s Q Test (P value)	Rucker’s Q Test (P value)	Egger Intercept (P value)	Distortion Test	Global Test	Normal Distribution
		IVW	MR-Egger	MR-Egger	Outliers	P value	P value
AAMA	BON	0.571	0.530	0.998	NA	0.543	0.679
AAMO		0.090	0.073	0.882	NA	0.109	0.768
AAMA	MON	0.626	0.651	0.213	NA	0.518	0.416
AAMO		0.642	0.626	0.435	NA	0.521	0.273

MR, mendelian randomization; AAMA, age at menarche; AAMO, age at menopause; BON, benign oesophageal neoplasia; MON, malignant oesophageal neoplasia.

**Figure 5:**
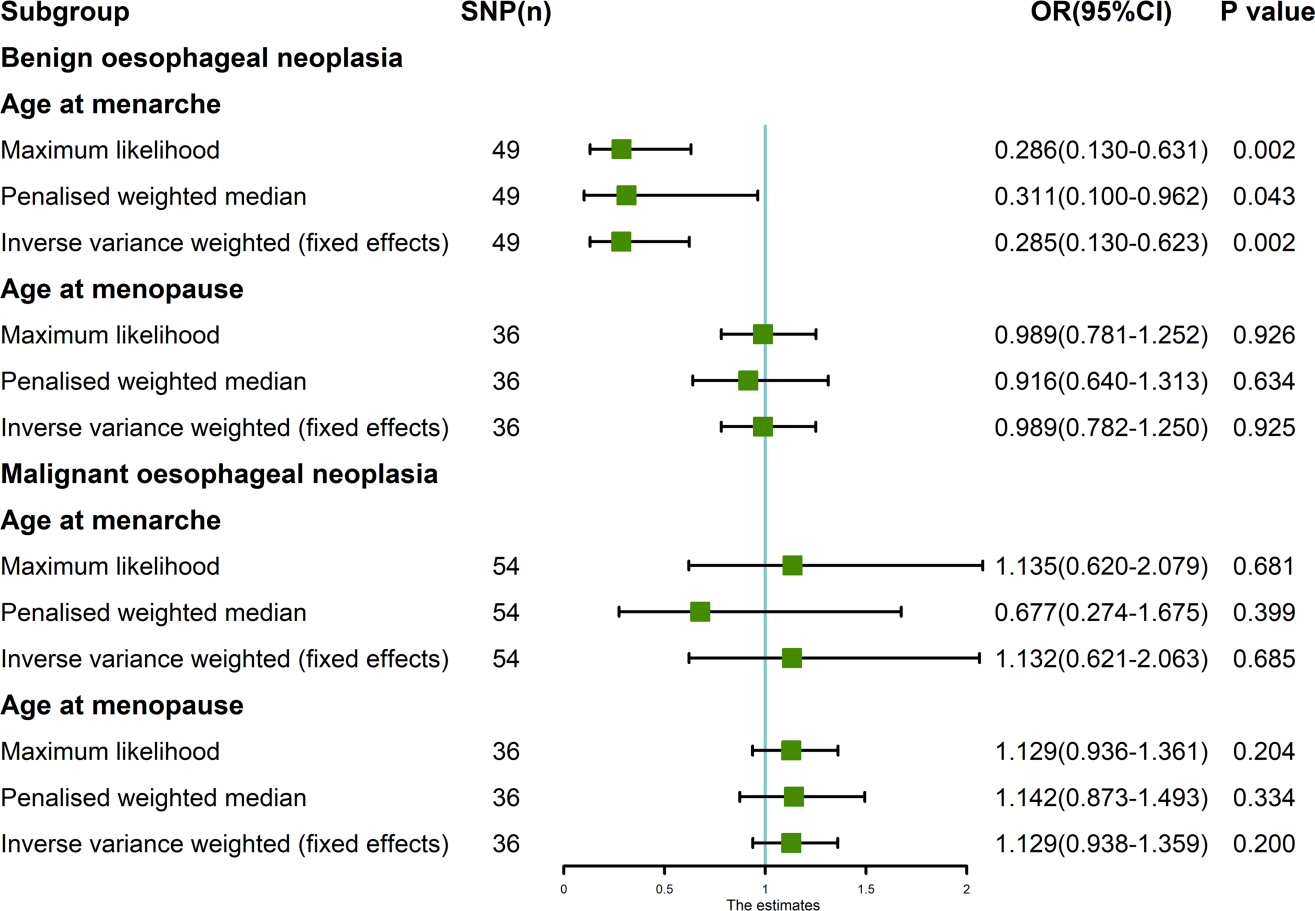
MR analysis between exposures (age at menarche and age at menopause) and outcomes (benign oesophageal neoplasia and malignant oesophageal neoplasia). Three methods used: maximum likelihood, penalized weighted median, and IVW (fixed effects). The blue vertical line represents OR = 1. The green square is the OR value of MR analysis result. The black line segment is the OR 95% confidence interval.

The random-effects IVW results showed that AAMO had no genetic causal relationship with BON (OR = 0.989 [95%CI: 0.755-1.296], *P* = 0.935) (**Figures 1**, **2B**). The MR analysis results of the MR Egger, weighted median, simple mode and weighted mode were consistent with random-effects IVW (**Figure 1**). The Cochran’s Q statistic of the MR-IVW method and Rucker’s Q statistic of the MR Egger method showed that the MR analysis of AAMO and BON had no heterogeneity (*P* > 0.05). The intercept test of MR Egger and global test of MR-PRESSO showed that the MR analysis of AAMO and BON had no horizontal pleiotropy (*P* > 0.05). The distortion test of MR-PRESSO analysis showed that the MR analysis of AAMO and BON had no outliers (**Table 1**). The leave-one-out analysis indicated that the MR analysis of AAMO and BON were not driven by a single SNP (**Figure 3B**), and the MR-RAPS analysis showed that the MR analysis between AAMO and BON was normally distributed (*P* > 0.05) (**Table 1**; **Figure 4B**). At the validation stage, maximum likelihood, penalized weighted median, and IVW (fixed effects) results showed that AAMA had a negative genetic causal relationship with BON (*P* < 0.05), while AAMO had no genetic causal relationship with BON (*P* > 0.05) (**Figure 5**).

### 3.3 MR analysis of AAMA, AAMO and MON

The random-effects IVW results showed that both AAMA (OR = 1.132 [95%CI: 0.621-2.063], *P* = 0.685) and AAMO (OR = 1.129 [95%CI: 0.938-1.359], *P* = 0.200) had no genetic causal relationship with MON. The results of MR Egger, weighted median, simple mode, and weighted mode were consistent with random-effects IVW (**Figures 1**, **2C, D**). The Cochran’s Q statistic of the MR-IVW method, and Rucker’s Q statistic of the MR Egger method, showed that the MR analysis of AAMA, AAMO and MON had no heterogeneity (*P* > 0.05). The intercept test of MR Egger and the global test of MR-PRESSO showed that the MR analysis of AAMA, AAMO and MON had no horizontal pleiotropy (*P* > 0.05). The distortion test of MR-PRESSO analysis showed that the MR analysis of AAMA, AAMO and MON had no outliers (**Table 1**). Leave-one-out analysis indicated that the MR analysis of AAMA, AAMO and MON were not driven by a single SNP (**Figures 3C, D**). MR-RAPS analysis showed that the MR analysis of AAMA, AAMO and MON were normally distributed (*P* > 0.05) (**Table 1**; **Figures 4C, D**). At the validation stage, maximum likelihood, penalized weighted median, and IVW (fixed effects) results showed that AAMA and AAMO had no genetic causal relationship with MON (*P* > 0.05) (**Figure 5**).

## 4 Discussion

We explored the genetic causality between exposures (AAMA and AAMO) and outcomes (BON and MON) by MR analysis based on GWAS summary data. We show that AAMA has a negative genetic causal relationship with BON, and that there is no genetic causal relationship between AAMA and MON, AAMO and BON, or AAMO and MON. However, this does not rule out correlations between them at other, non-genetic levels. Earlier AAMA has been reported to be associated with increased levels of several sex hormones, such as estrogen (51, 52). One study found that women with higher levels of free testosterone have a lower risk of ESCC and colorectal cancer (53). There have also been studies that link higher luteinizing hormone to a lower risk of ON in women (54). In a large number of previous studies, when discussing the relationship between AAMA and ON, it is basically because AAMA is closely related to sex hormones, and thus impacts ON through hormones.

Studies have found that estrogen can participate in the regulation of adipose tissue metabolism (55), and visceral fat is associated with imbalances in certain metabolic compounds and fat-related hormones (56, 57). Leptin is an adipokine closely related to body mass index, which can regulate food intake and energy consumption, and obese people often show hyperleptinemia due to leptin resistance (58). Moreover, obesity is an important part of the pathogenesis of ON (59, 60). We considered that the close correlation between AAMA and ON in previous studies may occur through AAMA-triggered changes related to sex hormones to affect the regulation of human obesity, ultimately impacting ON. A case-control study found that elevated blood leptin levels are strongly associated with an increased risk of Barrett’s esophagus (61). Moreover, studies have shown that leptin can directly induce the proliferation of a variety of human cells, including EC cells (62). In contrast, another study has indicated that ON has no clear association with most of the sex hormones, including sex hormone-binding globulin, dehydroepiandrosterone sulfate, testosterone, dihydrotestosterone, estradiol, progesterone, or free androgen (54). Therefore, still remains concerning the relationship between AAMA-related hormone changes and ON, our results might provide new evidence and ideas for these debates. Our results show that AAMA has a causal relationship with BON at the genetic level, but no causal relationship with MON. This conclusion does not conflict with the results of previous studies.

It has been reported that earlier AAMO is a risk factor for ESCC (25). In addition, a large data survey study also found that the risk of ON and gastric cancer could be related to the status of AAMO. In this study, it is noted that postmenopausal women have a higher risk of esophageal and stomach cancer than pre-or peri-menopausal women, and that among postmenopausal women, the younger the AAMO, the higher the risk of cancer (63). It was found that AAMO is associated with decreased levels of various sex hormones (64). Hormone therapy can effectively reduce the risk of EAC (22, 65). Based on the above discussion, it was not difficult to find that the relationship between AAMO and ON seems to be similar to that between AAMA and ON. Our results suggest that the association between AAMO and ON in previous studies may also be realized through hormonal pathways.

There are some limitations in this study. First, our study population involved only European women, so we need to be careful when generalizing our findings to other populations. In addition, the outcome data we used in our study contained both females and males. We expect that using a dataset that includes both males and females would have reduced the association strengths in this study, thus rendering our results conservative (28). Therefore, future MR studies may be warranted to verify our results in female-only samples.

## 5 Conclusion

AAMA and BON are causally related at the genetic level, but AAMA and MON are not. There is no genetic causal relationship between AAMO and BON or MON. Using MR, our study clarified the causal relationship between exposures (AAMA and AAMO) and outcomes (BON and MON), providing help for the future prevention, treatment and prognosis of ON. However, diseases are complex and changing, and we need to explore them further in the future, as well as explore their relationships in more detail.

## Data availability statement

The original contributions presented in the study are included in the article/**Supplementary Material**. Further inquiries can be directed to the corresponding authors.

## Author contributions

YP and YS designed the study. YS, YH, and YX analyzed the datasets and interpreted the results. MY provided software support. FW downloaded the data. YS wrote and edited the manuscript. MY, FW, and YP conducted scientific and quality control over the manuscript. YP provided the foundation and support. All authors contributed to the article and approved the submitted version.
