# Supplementary material for: Genetic causal relationship between age at menarche and benign oesophageal neoplasia identified by a Mendelian randomization study
Source: Front Endocrinol (Lausanne). 2023 Mar 21;14:1113765. doi: 10.3389/fendo.2023.1113765 (PMC10071044; doi:10.3389/fendo.2023.1113765)
Supplement: Supplementary file 6 [file Image_1.pdf]

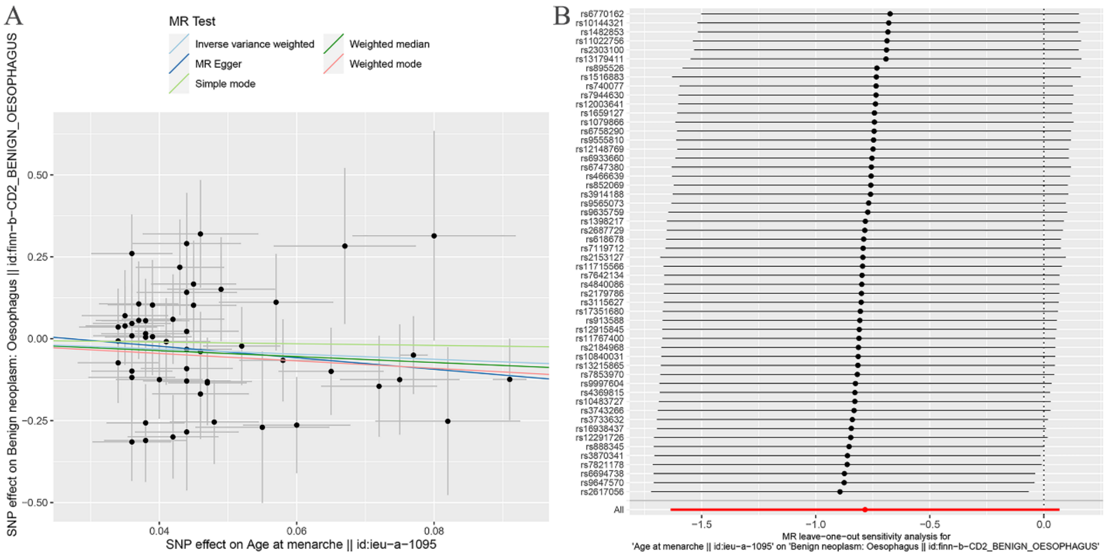

Supplementary Figure 1: Preliminary MR analysis of age at menarche and benign oesophageal neoplasia using 54 SNPs. A: Scatter plot of the MR analysis between age at menarche and benign oesophageal neoplasia; B: Leave one out analysis of the MR analysis between age at menarche and benign oesophageal neoplasia.
